# Supplementary material for: Content-rich biological network constructed by mining PubMed abstracts
Source: BMC Bioinformatics. 2004 Oct 8;5:147. doi: 10.1186/1471-2105-5-147 (PMC528731; doi:10.1186/1471-2105-5-147)
Supplement: Additional File 2 — The original results of the above study (non-essential files are deleted to keep the file size under the limit set by BMC bioinformatics). [file 1471-2105-5-147-S2.bz2 › chilibotAdditionalFile2/dip40/689ID9294424E18009/html/SECA_SECY.html]

 


 **SECA** and **SECY** 
  
Found 153 abstracts in PubMed, retrieved 40.  
 

 What does Google say? 
 PDF only 
| .edu only 

---

**Interactive relationship** (e.g. stimulation, inhibition, etc)

**Neutral relationship**- The interaction of the  **SecY**  proteins of S. griseus, S. lividans, and Escherichia coli, respectively, with the purified  **SecA**  protein of S. griseus was demonstrated for the first time by using ligand affinity blot assays.  Ref: 10542330 Biochim Biophys Acta, 1999
- A single amino acid substitution in  **SecY**  stabilizes the interaction with  **SecA** .  Ref: 10446151 J Biol Chem, 1999
- It is suggested that the C terminal  **SecY**  residues are required for  **SecA**  dependent translocation initiation.  Ref: 11914356 J Bacteriol, 2002
- The fifth and the sixth cytoplasmic regions C5 and C6 of  **SecY**  are important for the  **SecA**  driven preprotein translocation reaction.  Ref: 12351621 J Biol Chem, 2002
- CONCLUSIONS The Walker motif A regions of  **SecA**  are important for the  **SecA**   **SecY**  interaction that leads to the  **SecA**  conformational changes required for insertion into the SecYEG channel.  Ref: 11168585 Genes Cells, 2000
- **SecY**  I278C EG binds  **SecA**  with a higher affinity than normal SecYEG, in particular in the presence of ATP.  Ref: 10446151 J Biol Chem, 1999
- Northern blot analysis revealed a growth phase dependent  **secY**  expression supporting our previous findings for  **secA**  gene expression in S. griseus.  Ref: 10542330 Biochim Biophys Acta, 1999
- In concert with SecB,  **SecA**  targets the precursors to  **SecY**  and energizes their translocation by its ATPase activity.  Ref: 11051763 Prog Nucleic Acid Res Mol Biol, 2001
- **SecY**  F286Y supports only a low  **SecA**  binding affinity, preprotein translocation and  **SecA**  translocation ATPase activity.  Ref: 11755523 FEBS Lett, 2002
- Genetic analysis of  **SecA**   **SecY**  interaction required for spore development in Bacillus subtilis.  Ref: 10713435 FEMS Microbiol Lett, 2000
- We now show that a  **secY**  mutation, which compromises a functional  **SecY**   **SecA**  interaction, does not impair the SRP mediated integration of polytopic inner membrane proteins.  Ref: 10931878 J Cell Biol, 2000
- Characterization of a mutant form of  **SecA**  that alleviates a  **SecY**  defect at low temperature and shows a synthetic defect with  **SecY**  alteration at high temperature.  Ref: 10833277 J Biochem Tokyo , 2000
- The data also suggest that the conformation of the central region of  **SecA**  is important for the regulation of ATP hydrolysis and for the productive interaction of  **SecA**  with  **SecY** .  Ref: 10833277 J Biochem Tokyo , 2000
- Thus, this mutant provides an excellent system for genetic analysis of the  **SecY**   **SecA**  interaction.  Ref: 11168585 Genes Cells, 2000
- The former class of mutations, presumably with specific effects on the  **SecY**   **SecA**  interaction, clustered in two regions close to the Walker motif A sequences of the two ATP binding domains.  Ref: 11168585 Genes Cells, 2000
- We found that in this system  **SecA**  and ATP are dispensable for both the transfer of the nascent inner membrane protein FtsQ to  **SecY**  and its stable membrane insertion.  Ref: 10514469 J Biol Chem, 1999
- We introduced random mutations into a segment of Escherichia coli  **SecY**  within its cytoplasmic domain 5, which was shown previously to be important for the  **SecA**  dependent translocation activity.  Ref: 11309495 Proc Natl Acad Sci U S A, 2001
- Analysis and regulation of the  **secY**  gene 1 from Streptomyces griseus N2 3 11 and interaction of the  **SecY**  protein with the  **SecA**  protein.  Ref: 10542330 Biochim Biophys Acta, 1999
- E. coli  **SecA** , but not B. subtilis  **SecA** , supported efficient ATP dependent translocation of the E. coli precursor OmpA proOmpA into inner membrane vesicles containing the hybrid SecYEG complexes, if E. coli  **SecY**  and either E. coli SecE or E. coli SecG were present.  Ref: 10559168 J Bacteriol, 1999
- In Escherichia coli, three different types of proteins engage the  **SecY**  translocon of the inner bacterial membrane for translocation or insertion 1 polytopic membrane proteins which prior to their insertion into the membrane are targeted to the translocon using the bacterial signal recognition particle SRP and its receptor, 2 secretory proteins which are targeted to and translocated across the  **SecY**  translocon in a  **SecA**  and SecB dependent reaction and 3 membrane proteins with large periplasmic domains, requiring SRP for targeting and  **SecA**  for the translocation of the periplasmic moiety.  Ref: 12682042 J Biol Chem< MedlineTA>J Biol Chem, 1999
- This intramolecular regulator of ATP hydrolysis IRA mediates N C domain binding and acts as a molecular switch it suppresses ATP hydrolysis in cytoplasmic  **SecA**  while it releases hydrolysis in  **SecY**  bound  **SecA**  during translocation.  Ref: 10594836 Mol Microbiol, 1999
- As improved  **SecA**  binding to  **SecY**  was also observed with the prlA4 suppressor mutation, it may be a general mechanism underlying signal sequence suppression.  Ref: 10446151 J Biol Chem, 1999
- The antibodies inhibited translocation of OE33 but not OE23, indicating that cpSecY  [ **SECY** ]  comprises the protein conducting channel used in the  **SecA**  dependent pathway.  Ref: 10207046 J Biol Chem, 1999
- A mutation in  **secY**  that causes enhanced  **SecA**  insertion and impaired late functions in protein translocation.  Ref: 10852867 J Bacteriol, 2000

**Non-interactive relationship** (e.g. studied together, co-existance, homology, etc.)

- One set is more similar to  **SecA**  and  **SecY**  of Escherichia coli.  Ref: 12010500 Mol Microbiol, 2002
- Protein export in Escherichia coli is mediated by translocase, a multisubunit membrane protein complex with  **SecA**  as the peripheral subunit and the  **SecY** , SecE, and SecG proteins as the integral membrane domain.  Ref: 10074070 J Bacteriol, 1999
- This SRP, assisted by its receptor, FtsY, mediates the transfer to a common translocation site in the inner membrane that contains  **SecA** , SecG, and  **SecY** .  Ref: 10514469 J Biol Chem, 1999
- Crosslinking experiments showed that the hydrophilic region P1 of nascent membrane inserted Lep 100 mer was in the vicinity of  **SecA**  and  **SecY** .  Ref: 10913619 FEBS Lett, 2000
- In the gram positive bacterium Bacillus subtilis,  **SecA** ,  **SecY** , and SecE have been identified through genetic analysis.  Ref: 10074070 J Bacteriol, 1999
- Most bacterial species have a single copy of the genes encoding  **SecA**  and  **SecY** , which are essential for viability.  Ref: 12010500 Mol Microbiol, 2002
- Pulse chase experiments showed that significant amount of wild type RBP was processed in a  **secY**  mutant in the presence of azide  **SecA**  inhibitor.  Ref: 12810088 Biochem Biophys Res Commun, 2003
- For both substrates, the mRNA levels of  **secA** , secD, secE, secF, and  **secY**  genes were almost constant during the early and log phases, but showed a marked decrease at the beginning of the stationary phase followed by a full recovery of mRNA level in the late stationary phase.  Ref: 10696484 Can J Microbiol< MedlineTA>Can J Microbiol, 2003
- To test whether cpSecY  [ **SECY** ]  forms the protein conducting channel of the thylakoid membrane, translocation assays were conducted with the  **SecA**  dependent substrate OE33 and the  **SecA**  independent substrate OE23, in the presence and absence of antibodies raised against cpSecY  [ **SECY** ] .  Ref: 10207046 J Biol Chem, 1999
- Besides  **SecA**  and SecB, Escherichia coli cells possess a signal recognition particle SRP to target exported proteins to the  **SecY**  translocon.  Ref: 10619852 EMBO J, 2000
- Once targeted to  **SecY** , ribosome associated nascent chains of the hybrid protein remain untranslocated unless  **SecA**  is present.  Ref: 11101515 EMBO J, 2000
- Here, we use a cross linking approach to show that hydrophilic portions of a co translationally targeted inner membrane protein FtsQ are close to  **SecA**  and  **SecY** , suggesting that insertion takes place at the  **SecA**  Y interface.  Ref: 10675323 EMBO J, 2000
- Genes encoding  **SecA**  and  **SecY**  proteins, essential components of the Sec protein translocation system, were cloned from onion yellows phytoplasma, an unculturable plant pathogenic bacterium.  Ref: 11551069 Mol Plant Microbe Interact, 2001
- These data imply an important role for  **SecY**  transmembrane segment 7 in  **SecA**  binding.  Ref: 10446151 J Biol Chem, 1999
- The mass and structure of this SecYEG as well as the subunit stoichiometry of  **SecA**  and  **SecY**  in a soluble translocase precursor complex reveal that translocase consists of the  **SecA**  homodimer and four SecYEG complexes.  Ref: 10698927 EMBO J, 2000
- Translocation occurs at a multisubunit membrane bound enzyme termed translocase, which consists of a peripheral preprotein binding site and an ATPase domain termed  **SecA** , a core heterotrimeric integral membrane protein complex with  **SecY** , SecE and SecG as subunits, and an accessory integral membrane protein complex containing SecD and SecF.  Ref: 10066476 Curr Opin Microbiol, 1998
- These results indicate that SRP dependent integration and  **SecA**  SecB mediated translocation do not only represent two independent protein delivery systems, but also remain mechanistically distinct processes even at the level of the membrane where they engage different domains of  **SecY**  and different components of the translocon.  Ref: 10931878 J Cell Biol, 2000
- However, Streptococcus gordonii strain M99 encodes  **SecA**  and  **SecY**  homologues that are not required for viability or for the translocation of most exported proteins.  Ref: 12010500 Mol Microbiol, 2002
- Nascent Lep inserts into the Escherichia coli inner membrane in the vicinity of YidC,  **SecY**  and  **SecA** .  Ref: 10913619 FEBS Lett, 2000
- At least two other Gram positive species, Streptococcus pneumoniae and Staphylococcus aureus, encode two sets of  **SecA**  and  **SecY**  homologues.  Ref: 12010500 Mol Microbiol, 2002
- Bacterial protein translocation is mediated by translocase, a multisubunit membrane protein complex that consists of a peripheral ATPase  **SecA**  and a preprotein conducting channel with  **SecY** , SecE, and SecG as subunits.  Ref: 10559168 J Bacteriol, 1999
- prlA mutations in the gene encoding the  **SecY**  subunit of the membrane domain of the Escherichia coli preprotein translocase confer many phenotypes enhanced translocation rates, increased affinity for  **SecA** , diminished requirement for functional leader sequences, reduced proton motive force PMF dependence of preprotein translocation and facilitated translocation of preproteins with folded domains.  Ref: 10369667 EMBO J, 1999
- When isoleucine 278 in transmembrane segment 7 of the  **SecY**  subunit was replaced by a unique cysteine, SecYEG supported an increased preprotein translocation and  **SecA**  translocation ATPase activity, and allowed translocation of a preprotein with a defective signal sequence.  Ref: 10446151 J Biol Chem, 1999
- Previously, we have identified a cold sensitive  **secY**  mutation secY205, affecting the most carboxy terminal cytosolic domain, that did not allow an ATP dependent insertion of a  **SecA**  preprotein complex.  Ref: 11168585 Genes Cells, 2000
